# Formulation of a kit under Good Manufacturing Practices (GMP) for preparing [^111^In]In-BnDTPA-trastuzumab-NLS injection: a theranostic agent for imaging and Meitner-Auger Electron (MAE) radioimmunotherapy of HER2-positive breast cancer

**DOI:** 10.1186/s41181-022-00186-9

**Published:** 2022-12-21

**Authors:** Conrad Chan, Vanessa Prozzo, Sadaf Aghevlian, Raymond M. Reilly

**Affiliations:** 1 Department of Pharmaceutical Sciences, University of Toronto, Toronto, ON Canada; 2 Princess Margaret Cancer Centre, Toronto, ON Canada; 3 Department of Medical Imaging, University of Toronto, Toronto, ON Canada; 4 Joint Department of Medical Imaging, University Health Network, Toronto, ON Canada

**Keywords:** Trastuzumab, ^111^in, Nuclear Translocation Sequence (NLS), Kit, Good Manufacturing Practices (GMP), Meitner-Auger electrons, Imaging, Radioimmunotherapy

## Abstract

**Background:**

^111^In[In]-BnDTPA-trastuzumab-NLS is a radiopharmaceutical with theranostic applications for imaging and Meitner-Auger electron (MAE) radioimmunotherapy (RIT) of HER2-positive breast cancer (BC). Nuclear localization sequence (NLS) peptides route the radiopharmaceutical to the nucleus of HER2-positive BC cells following receptor-mediated internalization for RIT with subcellular range MAEs. The γ-photons emitted by ^111^In permit tumour imaging by SPECT. Our aim was to formulate a kit under Good Manufacturing Practices conditions to prepare ^111^In[In]-BnDTPA-trastuzumab-NLS injection for a first-in-human clinical trial.

**Results:**

Trastuzumab was derivatized with p-SCN-BnDTPA to introduce Bn-DTPA for complexing ^111^In, then modified with maleimide groups for conjugation to the thiol on cysteine in NLS peptides [CGYGPKKKRKVGG]. BnDTPA-trastuzumab-NLS (5 mg in 1.0 mL of 0.05 M ammonium acetate buffer, pH 5.5) was dispensed into unit dose sterile glass vials to produce kits for labeling with 100–165 MBq of ^111^In[In]Cl_3_. The kits met specifications for protein concentration (4.5–5.5 mg/mL), volume (0.95–1.05 mL), pH (5.5–6.0), appearance (clear, pale-yellow, particulate-free), BnDTPA substitution level (2.0–7.0 BnDTPA/trastuzumab), purity and homogeneity (SDS-PAGE and SE-HPLC), ^111^In labeling efficiency (> 90%), binding to HER2-positive SK-BR-3 human breast cancer cells (K_a_ = 1–8 × 10^8^ L/mmol; B_max_ = 0.5–2 × 10^6^ sites/cell), NLS peptide conjugation (upward band shift on SDS-PAGE), sterility (USP Sterility Test) and endotoxins (USP Bacterial Endotoxins Test). ^111^In-BnDTPA-trastuzumab-NLS injection met specifications for pH (5.5–6.5), radiochemical purity (≥ 90%), radionuclide purity (≥ 99%), appearance (clear, colourless, particle-free) and sterility (retrospective USP Sterility Test). Kits were stable stored at 2–8 °C for up to 661 days (d) meeting all key specifications. Protein concentration remained within or just slightly greater than the specification for up to 139 d. ^111^In[In]-BnDTPA-trastuzumab-NLS injection was stable for up to 24 h. An expiry of 180 d was assigned for the kits and 8 h for the final radiopharmaceutical.

**Conclusion:**

A kit was formulated under GMP conditions for preparing ^111^In[In]-BnDTPA-trastuzumab-NLS injection. This radiopharmaceutical was safely administered to 4 patients with HER2-positive BC to trace the uptake of trastuzumab into brain metastases before and after MRI-guided focused ultrasound (MRIg-FUS) by SPECT imaging.

## Background

Human epidermal growth factor receptor-2 (HER2) positive breast cancer (BC) accounts for 15–20% of cases of BC and was previously considered a poor prognosis subtype (Burstein 2005). However, the introduction of HER2-targeted therapies such as trastuzumab (Herceptin; Roche), pertuzumab (Perjeta; Roche) and the antibody–drug conjugate (ADC), trastuzumab-emtansine (T-DM1, Kadcyla; Roche) and the small molecule HER2/EGFR tyrosine kinase inhibitor (TKI), lapatinib (Tykerb; Novartis) have improved the outcome of patients with HER2-positive BC, including those with metastatic disease (Swain et al. 2015; Verma et al. 2012; Geyer et al. 2006). These HER2-targeted therapies are often combined with a taxane or other chemotherapy (Martinez-Saez and Prat 2021). In addition, several new targeted agents for treating HER2-positive BC have recently been introduced including the TKI tucatinib (Tukysa; Cascadian Therapeutics) (Murthy et al. 2020) and fam-trastuzumab deruxtecan-nxki (Enhirtu, Roche), an ADC that links trastuzumab to a potent topoisomerase I inhibitor (Martinez-Saez and Prat 2021).

Radioimmunotherapeutic agents (RIT) are analogous to ADCs and link monoclonal antibodies (mAbs) to radionuclides that emit β-particles (e.g. ^131^I, ^177^Lu, ^90^Y), α-particles (e.g. ^225^Ac, ^213^Bi, ^211^At) or Meitner-Auger electrons (MAEs; e.g. ^111^In, ^125^I, ^67^Ga) for radiation treatment of tumours (Aghevlian et al. 2017; Ku et al. 2019). Trastuzumab labeled with the β-particle emitter, ^177^Lu was cytotoxic in vitro to HER2-positive human SK-BR-3 and MDA-MB-453 human BC cells and SK-OV-3 ovarian cancer cells (Sharma et al. 2020). Treatment of athymic mice with intraperitoneal (i.p.) HER2-positive LS-174 T human colon cancer xenografts with i.p. administered ^177^Lu-labeled trastuzumab prolonged survival more than 12-fold compared to untreated mice (Ray et al. 2011). ^177^Lu-labeled trastuzumab administered to patients with HER2-positive BC localized in primary and metastatic tumors and these were detected by single photon emission computed tomography/computed tomography (SPECT/CT) illustrating its potential for imaging and RIT of HER2-positive BC (Bhusari et al. 2017). Our group reported that a bispecific RIT agent composed of trastuzumab Fab linked through a polyethylene glycol (PEG_24_) spacer to epidermal growth factor (EGF) labeled with ^177^Lu was cytotoxic in vitro to human BC cells expressing HER2 or epidermal growth factor receptors (EGFR) or both receptors (Razumienko et al. 2016). This agent was effective for RIT of HER2 and EGFR-positive MDA-MB-231/H2N tumours in athymic mice but hematopoietic toxicity limited the dose that could be safely administered. RIT of solid tumours in humans with β-particle emitters has similarly proven dose-limited by off-target hematopoietic toxicity (Larson et al. 2015) due to the long (several millimeters) path length of β-particles (cross-fire effect) (Richman et al. 2005). In addition, β-particles have very low linear energy transfer (LET = 0.1–1 keV/μm) that makes them theoretically less potent than shorter range (28–100 μm) and higher LET α-particles (LET = 50–230 keV/μm) or nanometer-micrometer range MAEs (LET = 4–26 keV/μm) for killing cancer cells (Aghevlian et al. 2017).

Trastuzumab labeled with α-particle emitting, ^225^Ac administered by intraperitoneal (i.p.) injection was effective for RIT of HER2-positive SK-OV-3 human ovarian cancer tumours in athymic mice (Borchardt et al. 2003). ^225^Ac-labeled trastuzumab was also effective for treatment of SUM225 ductal carcinoma in situ of the breast tumours in NCG mice after intraductal injection (Yoshida et al. 2016). However, intravenous (i.v.) injection of ^225^Ac-labeled trastuzumab in these studies caused hematopoietic system toxicity, possibly due to irradiation of bone marrow stem cells by the 28–100 μm range α-particles emitted by circulating ^225^Ac-labeled trastuzumab perfusing the bone marrow (cross-fire effect). In addition, the ^213^Bi decay product of ^225^Ac poses a risk for renal toxicity (Yoshida et al. 2016).

MAE-emitting radionuclides are an alternative to α-particle emitters for RIT since they similarly exhibit high LET, but their cytotoxic effects are restricted to cells that bind and internalize the radioimmunoconjugates (RICs) due to the subcellular range of the electrons. There is no cross-fire effect from MAE-emitting radionuclides, which greatly reduces off-target toxicity (e.g. hematopoietic toxicity) compared to longer range β-emitters such as ^177^Lu (Ku et al. 2019; Aghevlian et al. 2017). Moreover, MAE-emitters decay to stable elements which avoids toxicity from radioactive daughter products, a challenge for α-particle emitters such as ^225^Ac. MAEs kill cancer cells by causing oxidative damage to the cell membrane (Paillas et al. 2016) or by inflicting lethal DNA double-strand breaks (Ku et al. 2019). DNA damage from MAEs is the most potent and may be maximized by conjugation of the mAbs to peptides that incorporate nuclear translocation sequences (NLS) or by targeting a receptor that harbours an endogenous NLS. NLS are short sequences of 4 or more cationic amino acids [arginine (R) or lysine (K)] that bind to importin-α which forms a complex with importin-β and mediates active transport of proteins across the nuclear pore complex (NPC) (Costantini et al. 2008b). Only proteins with molecular weight (MW) < 40–45 kDa are able to diffuse across the NPC due to the size of the pores (25–30 nm). A classical NLS is the Simian Virus-40 (SV-40) large T-antigen NLS [^126^PKKKRKV^132^] but many peptide growth factors and their receptors harbour endogenous NLS (Costantini et al. 2008b).

We modified trastuzumab with diethylenetriaminepentaacetic acid (DTPA) to complex the MAE-emitter, ^111^In (t_1/2_ = 2.8 d) and subsequently conjugated these RICs to 13-mer peptides [CGYG*PKKKRKV*GG] that harboured the NLS of SV-40 large T-antigen (italics) to promote nucleus uptake after HER2-mediated internalization into BC cells (Costantini et al. 2007). Treatment of athymic mice with subcutaneous (s.c.) HER2-positive MDA-MB-361 human BC xenografts with a single amount of ^111^In[In]-DTPA-trastuzumab-NLS (9.25 MBq; 4 mg/kg) administered by i.p. injection inhibited tumour growth by fourfold compared to untreated mice and did not cause any normal tissue toxicity, including to the hematopoietic system (Costantini et al. 2010a). Two amounts of ^111^In[In]-DTPA-trastuzumab-NLS (9.25 MBq; 4 mg/kg) separated by two weeks prolonged survival by 1.7-fold compared to untreated mice (Costantini et al. 2010a). Furthermore, ^111^In[In]-DTPA-trastuzumab-NLS killed trastuzumab-resistant HER2-positive BC cells in vitro (Costantini et al. 2008a). These cytotoxic effects of ^111^In[In]-DTPA-trastuzumab-NLS were radiosensitized by methotrexate, paclitaxel or doxorubicin (Costantini et al. 2010b).

Based on these encouraging preclinical results, our aim in the current study was to formulate a kit for preparing ^111^In[In]-BnDTPA-trastuzumab-NLS injection under Good Manufacturing Practices (GMP) conditions to enable advancement to a first-in-human clinical trial. The p-isothiocyanate ester of DTPA (p-SCN-BnDTPA) was used rather than DTPA dianhydride employed prevously to modify trastuzumab with DTPA (Costantini et al. 2007), since this bifunctional chelator provides more stable complexes with ^111^In and minimizes cross-linking of trastuzumab molecules, which may occur due to the two reactive groups present in DTPA dianhydride (Brechbiel et al. 1986). In addition to the MAE emissions, ^111^In emits γ-photons [Eγ = 171 keV (90.7%) and 245 keV (94.1%)] that permit imaging by single photon emission computed tomography (SPECT). Thus, for this first-in-human clinical trial, we formulated a kit for labeling 5 mg of DTPA-trastuzumab-NLS with 111–165 MBq of ^111^In to assess the tumour and normal tissue uptake of this radiopharmaceutical in humans by SPECT. These mass and activity amounts were selected based on those previously reported for SPECT imaging of HER2-positive BC in humans using ^111^In[In]DTPA-trastuzumab (100–150 MBq; 5 mg) (Perik et al. 2006).

^111^In[In]-BnDTPA-trastuzumab-NLS injection prepared from the kit described here was recently administered safely for the first time to 4 patients with HER2-positive BC to trace the uptake of trastuzumab into brain metastases, prior to and after application of MRI guided focused ultrasound (MRIg-FUS) (Meng et al. 2021). MRIg-FUS is an interventional technique that transiently and spatially disrupts the blood–brain-barrier (BBB), facilitating penetration of trastuzumab into metastatic lesions in the brain (Park et al. 2012). There were no adverse reactions associated with administration of the radiopharmaceutical. This study revealed that MRIg-FUS significantly improved the delivery of trastuzumab into brain metastases by as much as 4.5-fold. These results further suggest that ^111^In[In]-BnDTPA-trastuzumab may be a promising MAE-emitting RIT agent for treating brain metastases in patients with HER2-positive BC by employing MRIg-FUS-enhanced delivery into the brain. Thus, ^111^In-BnDTPA-trastuzumab-NLS is a theranostic agent with promising application for imaging and MAE RIT of HER2-positive BC.

## Methods

### Trastuzumab, NLS peptides and p-SCN-BnDTPA

Trastuzumab (Herceptin^®^; Hoffman La Roche, Mississauga, ON, Canada) was purchased from the Princess Margaret Cancer Centre (Toronto, ON, Canada) hospital pharmacy and reconstituted with the supplied Bacteriostatic Water for Injection, USP to 21 mg/mL following the manufacturer's directions. Trastuzumab identity and purity were determined by sodium dodecyl sulfate–polyacrylamide gel electrophoresis (SDS-PAGE), Western blot and size-exclusion high performance liquid chromatography (SE-HPLC). We previously used these analytical methods to study the stability of trastuzumab for short term storage at room temperature (RT) or at 2–8 °C (Chan et al. 2020). For SDS-PAGE, ~ 2 μg of trastuzumab was electrophoresed on a 4–20% Tris–HCl gradient mini-gel (BioRad, Hercules, CA, USA) under reducing (dithiothreitol, DTT) and non-reducing conditions. Protein bands were stained with Coomassie R-250 Brilliant Blue. Broad range molecular weight (MW) standards were electrophoresed to calibrate the gel. Western blot was performed by transferring electrophoresed proteins onto a polyvinylidene fluoride (PVDF) membrane (Immun-Blot, BioRad, Hercules, CA, USA) and probing with goat anti-human Fab-specific horseradish peroxidase (HRP) immunoconjugates (Sigma-Aldrich, St. Louis, MO, USA). Bands were developed with diamidobenzidine/0.03% H_2_O_2_ (Sigma-Aldrich). Trastuzumab (~ 10 μg) was analysed for purity and homogeneity by SE-HPLC (Agilent Technologies, Santa Clara, CA) on a BioSep SEC-s4000 column (Phenomenex, Torrance, CA, USA) eluted with 100 mM NaH_2_PO_4_ buffer, pH 7.0 at a flow rate of 0.8 mL/min with UV detection at 280 nm. Peptides harbouring the nuclear translocation sequence (NLS; italics) of SV-40 large T-antigen [CGYG*PKKKRKV*GG] (Costantini et al. 2008b) were synthesized commercially by Bio Basic, Inc. (Markham, ON, Canada). The purity of the NLS peptides was > 98.6% measured by reversed-phase HPLC and mass spectroscopy. A certificate of actual lot analysis (COA) was obtained from the manufacturer which included HPLC analysis and a mass spectrum confirming the expected MW (1,419 Da). The identity of NLS peptides was determined by amino acid composition (SPARC Biocentre/Advanced Protein Technology Centre, Hospital for Sick Children, Toronto, ON, Canada). S-2-(4-isothiocyanatobenzyl)-diethylenetriamine pentaacetic acid (p-SCN-BnDTPA; purity ≥ 94%) was purchased from Macrocyclics (Dallas, TX, USA). A COA was obtained and the identity of p-SCN-BnDTPA was confirmed by ^1^H NMR (Varian Mercury 400 MHz) and by obtaining a UV–visible spectrum (200–900 nm) dissolved in 0.1 M sodium bicarbonate (NaHCO_3_) buffer, pH 8.2 (0.3 mg/mL) using a spectrophotometer (Bio Tek, Winooski, VT, USA).

### Other raw materials, reagents and vials

Ammonium acetate, ACS (NH_4_CO_2_CH_3_), sodium phosphate dibasic heptahydrate, USP (Na_2_HPO_4_ ·7H_2_O), polysorbate 20 (Tween 20^®^) and NaHCO_3_, USP were obtained from Sigma-Aldrich and tested for identity by USP methods. Sulfo-SMCC (sulfosuccinimidyl 4-(N-maleimidomethyl)cyclohexane-1-carboxylate) was purchased from Fisher Scientific (Ottawa, ON, Canada). All other chemicals and reagents were purchased from suppliers in ACS analytical grade (purity ≥ 95%) or were EP grade. Radiochemical quality ^111^In[In]Cl_3_ (> 3.7 GBq/mL; < 0.1% ^114m^In and ^65^Zn) was purchased from BWXT Medical Ltd. (Ottawa, ON, Canada). Sterile Water for Irrigation, USP and Sodium Chloride for Irrigation, USP were purchased from Baxter (Toronto, ON, Canada). COA were obtained for all non-pharmacopoeial raw materials. Sterile, apyrogenic 5 mL and 30 mL Type I glass vials with a grey rubber septum and aluminum seal meeting USP specifications were purchased from Omega Laboratories (Montreal, QC, Canada).

### Pharmaceutical quality buffers

Sterile 0.1 M NaHCO_3_ buffer, pH 8.2 in Sodium Chloride for Injection, USP and 0.05 M NH_4_CO_2_CH_3_ buffer, pH 5.5 and 0.1 M Na_2_HPO_4_ buffer in Sterile Water for Injection USP, pH 7.3 were prepared as previously reported (Lam et al. 2015). Trace metals were removed from all buffers by passing through a 10 mL column filled with Chelex-100 cation exchange resin (BioRad) followed by re-adjustment of the pH with sterile 1 N HCl, 1 N NaOH or glacial acetic acid USP. Buffers were sterilized by filtration through a 0.22 μm Millex GV filter (Sigma-Aldrich) into 30 mL glass vials and stored at 2–8 °C. All buffers were tested for sterility by the USP Sterility Test. The concentration of NaHCO_3_ was assayed by titration with 0.1 N sulfuric acid according to the USP method. The concentration of Na_2_HPO_4_ was determined by a colorimetric assay using ammonium molybdate and stannous chloride (Truong and Meyer 1929). The concentration of NH_4_CO_2_CH_3_ was not assayed due to unavailability of a method but instead was calculated by dividing the weight of NH_4_CO_2_CH_3_ used by the volume of the solution. Clarity and colour of the buffers were assessed against a light and dark background.

### Kit formulation

Kits for the preparation of ^111^In[In]-BnDTPA-trastuzumab-NLS injection were formulated under GMP conditions in a Class II Type A2 biosafety cabinet (Model 425–400; NuAire, Plymouth, MN, USA). Trastuzumab (Herceptin, Roche; 3.6 mL; 75.6 mg) reconstituted in Bacteriostatic Water for Injection, USP was buffer exchanged into 0.1 M NaHCO_3_ buffer, pH 8.2 using a 15 mL Amicon Ultra-15 centrifugal filter device [molecular weight cut-off (MWCO) = 30 kDa] (MilliporeSigma, Burlington, MA, USA). Briefly, trastuzumab was dispensed into the device and diluted to 12.0 mL with 0.1 M NaHCO_3_ buffer, pH 8.2. The device was centrifuged for 10 min at 5,000 × g at 18 °C, until ~ 2 mL of solution was retained. This solution was re-diluted to 12 mL with 0.1 M NaHCO_3_ buffer, pH 8.2 and centrifuged again. This was repeated a total of 4 times. The retentate was recovered and a sample of 2 μL was diluted 1:40 to determine the concentration of IgG by measuring the absorbance at 280 nm using the extinction coefficient 1.5 mL mg ^−1^ cm^−1^ for IgG. The trastuzumab concentration was adjusted to 16 mg/mL with 0.1 M NaHCO_3_ buffer, pH 8.2 and a volume of ~ 5.0 mL. Trastuzumab was then reacted with a 15-fold molar excess of p-SCN-BnDTPA (10 mg/mL freshly prepared in 0.1 M NaHCO_3_ buffer, pH 8.2) in a sterilized 10 mL glass Reacti-Vial^®^ (ThermoFisher Scientific, Waltham, MA) at RT for 1 h. Duplicate samples (12 μL) of the conjugation reaction were removed for subsequent determination of conjugation efficiency (CE) in order to estimate the number of BnDTPA conjugated to trastuzumab (see Quality Control Testing of Kits). The reaction mixture was transferred to an Amicon Ultra-15 centrifugal filter device (MWCO = 30 kDa) and re-diluted to 12 mL with 0.1 M Na_2_HPO_4_ buffer, pH 7.3. The device was centrifuged at 5,000 × g at 18 °C for 10 min until ~ 2 mL was retained. This solution was re-diluted to 12 mL with 0.1 M Na_2_HPO_4_ buffer, pH 7.3 and centrifuged again. This was repeated a total of 16 times to completely remove unconjugated p-SCN-BnDTPA. Purified BnDTPA-trastuzumab was recovered in ~ 8 mL of 0.1 M Na_2_HPO_4_ buffer, pH 7.3 and transferred to a pre-weighed 15 mL sterile polypropylene tube (Sarstedt, Nümbrecht, Germany). The tube was re-weighed to calculate the volume of recovered solution by difference, assuming 1 g = 1 mL. A sample (2 μL) was removed and diluted 40-fold with 0.1 M Na_2_HPO_4_ buffer, pH 7.3 and the absorbance was measured at 280 nm to calculate the concentration of BnDTPA-trastuzumab. Finally, BnDTPA-trastuzumab was diluted to 6.0 mg/mL with 0.1 M Na_2_HPO_4_ buffer, pH 7.3.

BnDTPA-trastuzumab was then reacted with a fivefold molar excess of Sulfo-SMCC (10 mg/mL freshly dissolved in 0.1 M Na_2_HPO_4_ buffer, pH 7.3) in a 10 mL sterilized glass Reacti-vial at RT for 1 h to introduce maleimide functional groups for reaction with the thiol on cysteine in NLS peptides. Excess unconjugated Sulfo-SMCC was removed by transferring the reaction mixture to an Amicon Ultra-15 centrifugal filter device (MWCO = 30 kDa), diluting to 12 mL with 0.1 M Na_2_HPO_4_ buffer, pH 7.3 and centrifuging the device at 5000 × g at 18 °C for 10 min. This retained solution was diluted again to 12 mL with 0.1 M Na_2_HPO_4_ buffer, pH 7.3 and the device centrifuged again. This was repeated a total of 10 times. Purified maleimide-functionalized BnDTPA-trastuzumab solution was recovered into a pre-weighed 15 mL sterile polypropylene tube and the volume of the solution was determined by difference, assuming 1 g = 1 mL. A sample (2 μL) was diluted 40-fold with 0.1 M Na_2_HPO_4_ buffer, pH 7.3 and the absorbance measured at 280 nm to determine the concentration of maleimide-functionalized BnDTPA-trastuzumab. The concentration was adjusted to 5.0 mg/mL with 0.1 M Na_2_HPO_4_ buffer, pH 7.3.

Maleimide-functionalized BnDTPA-trastuzumab was reacted with a 60-fold molar excess of NLS peptides (20 mg/mL in 0.1 M Na_2_HPO_4_ buffer, pH 7.3) in a 10 mL sterilized glass Reacti-vial at 4 °C overnight. Unconjugated NLS peptides were removed by transferring the reaction mixture to an Amicon Ultra-15 centrifugal filter device and diluting with 0.05 M NH_4_CO_2_CH_3_ buffer, pH 5.5. The solution was centrifuged at 5000 × g for 10 min, the filtrate discarded and the retained solution diluted again with 0.05 M NH_4_CO_2_CH_3_ buffer, pH 5.5. This was repeated a total of 15 times. The concentration of recovered purified BnDTPA-trastuzumab-NLS was determined by measuring the absorbance at 280 nm and the solution was diluted to a final concentration of 5.0 mg/mL with 0.05 M NH_4_CO_2_CH_3_ buffer, pH 5.5. Tween 20^®^ surfactant (Sigma-Aldrich) was added into the BnDTPA-trastuzumab-NLS solution to a final concentration of 0.1% to prevent protein aggregation (Strickley and Lambert 2021). The BnDTPA-trastuzumab-NLS solution was drawn up in a 5 mL sterile syringe with an 18G × 1½” needle (Becton-Dickenson, Franklin Lakes, NJ, USA) and sterilized by filtration through a 0.22 μm Millex GV low protein-binding filter (Sigma-Aldrich) into a 30 mL sterile glass vial with grey butyl rubber septum and aluminum seal. The integrity of the sterilizing filter was checked by the bubble test. Then 1.0 mL aliquots (5.0 mg of BnDTPA-trastuzumab-NLS) were drawn up using a 1 cc U-1000 insulin syringe with 28G × ½” gauge needle (Becton-Dickenson) and aseptically dispensed into sterile 5-mL glass vials to produce unit-dose kits. Kits were stored in the refrigerator at 2–8 °C.

### Quality control testing of kits

Kits were tested against specifications for protein concentration (4.5–5.5 mg/mL), volume (0.95–1.05 mL), pH (5.5–6.0), appearance (clear, pale-yellow, particulate-free), BnDTPA substitution level (2.0–7.0 BnDTPA/trastuzumab), purity and homogeneity (SDS-PAGE and SE-HPLC), ^111^In labeling efficiency (> 90%), binding to HER2-positive SK-BR-3 human breast cancer cells (K_a_ = 1–8 × 10^8^ L/mmol; B_max_ = 0.5–2 × 10^6^ sites/cell), NLS peptide conjugation (upward shift in the protein band on SDS-PAGE compared to BnDTPA-trastuzumab), sterility (USP Sterility Test) and endotoxins (USP Bacterial Endotoxins Test). Protein concentration was determined by measuring the absorbance at 280 nm. The volume in each kit vial was measured by the difference in weight of the vial before and after dispensing 1.0 mL of BnDTPA-trastuzumab-NLS solution into the vial, assuming 1 g = 1 mL. The pH was measured by spotting a sample on pH 4.5–7.5 range pH paper (pHydrion^®^, Micro Essential Laboratory, Brooklyn, NY, USA). Appearance was inspected by examining the colour, clarity and presence of any particles against a light or dark background.

SDS-PAGE was performed by electrophoresing ~ 2 μg on a 4–20% Tris–HCl gradient mini-gel (BioRad) under reducing (DTT) and non-reducing conditions. Protein bands were stained with Coomassie R-250 Brilliant Blue. The gel was calibrated by electrophoresing broad range MW standards. The stained SDS-PAGE gel was imaged on a BioRad ChemiDoc Imaging System (Mississauga, ON, Canada) and the relative density of each band determined using BioRad Image Lab (ver 6.0) software. The number of NLS peptides conjugated to trastuzumab was estimated by comparing the approximate MW of electrophoresed BnDTPA-trastuzumab-NLS versus BnDTPA-trastuzumab, assuming that each NLS peptide has a MW = 1,419 Da. SE-HPLC was performed on a BioSep SEC-s4000 column (Phenomenex) eluted with 100 mM NaH_2_PO_4_ buffer, pH 7.0 at a flow rate of 0.8 mL/min with UV detection at 280 nm (Agilent Technologies). To avoid sticking of the immunoconjugates to the matrix of the column, the cationic charges on NLS peptides were masked prior to analysis by reaction with a 50-fold molar excess of citraconic anhydride (Sigma-Aldrich, 1.25 g/mL in H_2_O) for 2 h at RT. Excess citraconic acid was removed by ultrafiltration on an Amicon Ultra-0.5 mL centrifugal filter device, repeated 8 times and a 20 μL sample (~ 15 μg) of the recovered BnDTPA-trastuzumab-NLS solution was chromatographed. The manufacturer of the BioSep SEC-s4000 column (Phenomenex) indicates that molecules with MW ranging from 244 Da (uridine) to 900 kDa (IgM) are well-separated. Trastuzumab (MW = 170 kDa), NLS peptides (MW = 1,419 Da) and unconjugated p-SCN-BnDTPA (MW = 649.9 Da) are separated by this column.

BnDTPA substitution level (moles BnDTPA/mole trastuzumab) was determined by trace-labeling duplicate 10 μL samples (~ 140 μg) of the unpurified reaction mixture (retained earlier) with ~ 1.0 MBq (~ 3.4 μL) of ^111^InCl_3_ (BWXT Medical Ltd.) mixed with ~ 10 μL of 0.05 M NH_4_CO_2_CH_3_ buffer, pH 5.5 in a 1.5 mL Eppendorf tube for 2 h at RT. The sample was analysed by instant thin layer-silica gel chromatography (ITLC-SG; Agilent Technologies) developed in 0.1 M sodium citrate buffer, pH 5.0 to measure the fraction of ^111^In[In]-BnDTPA-trastuzumab (R_f_ = 0.0) and ^111^In-BnDTPA (R_f_ = 1.0). The fraction of ^111^In-BnDTPA-trastuzumab was then multiplied by the 15-fold molar excess of p-SCN-BnDTPA used in the reaction to calculate the number of moles of BnDTPA/trastuzumab. The labeling efficiency (LE) of the kits with ^111^In was determined by aseptically decapping a single kit vial in a Class II Type A2 biosafety cabinet (Model 425–400; NuAire) and adding 100–165 MBq (~ 19 μL) of ^111^InCl_3_ (BWXT Medical Ltd.) mixed with 38 μL of 0.05 M NH_4_CO_2_CH_3_ buffer, pH 5.5 using a digital pipette with sterile tip and incubating at RT for 1–2 h. The percentage of ^111^In[In]-BnDTPA-trastuzumab-NLS was determined by ITLC-SG.

The binding of ^111^In[In]-BnDTPA-trastuzumab-NLS to HER2-positive SK-BR-3 cells was determined by a direct (saturation) binding assay as previously reported (Lam et al. 2015). Briefly, total binding (TB) was determined by incubating increasing concentrations [0.07–300 nmoles/L) of ^111^In[In]-BnDTPA-trastuzumab-NLS in phosphate-buffered saline (PBS) with 1 × 10^6^ SK-BR-3 cells in 1.5 mL Eppendorf tubes at 4 °C for 3.5 h with gentle shaking. The tubes were centrifuged at 2,000 rpm for 5 min to recover the cell-bound (pellet) and free (supernatant) radioactivity which was then measured in a γ-counter (Model Wallac Wizard 1480, PerkinElmer, Waltham, MA, USA). The assay was repeated in the presence of an excess (15.2 μmoles/L) of unlabeled trastuzumab to determine non-specific binding (NSB). Specific binding (SB) was calculated by subtraction of NSB from TB and plotted vs. the concentration of free ^111^In[In]-BnDTPA-trastuzumab-NLS (nmoles/L). The curve was fitted to a one-site receptor-binding model by Prism Ver. 4.0 software (GraphPad, San Diego, CA, USA) and the affinity constant (K_a_) and maximum number of binding sites per cell (B_max_) were calculated.

Kits were tested by the USP Sterility Test at the Microbiology Laboratory at Mount Sinai Hospital (Toronto, ON, Canada). Kits were tested for endotoxins by the USP Bacterial Endotoxins Test using the QCL-1000 Endpoint Chromogenic LAL Assay (Lonza, Walkersville, MD, USA). The stability of kits stored at 2–8 °C was assessed at selected intervals up to 661 d to establish an expiry by re-testing against specifications for protein concentration, pH, appearance, purity and homogeneity, labeling efficiency with ^111^In and HER2-binding properties. Sterility and endotoxins were not re-tested as it was expected that these would be maintained in storage since the kit solution was maintained sterile and endotoxin-free by packaging in sealed sterile, apyrogenic 5 mL glass vials.

### ^111^In[In]-BnDTPA-trastuzumab-NLS injection

^111^In[In]-BnDTPA-trastuzumab-NLS injection was prepared by aseptically decapping a single kit vial in a Class II Type A2 biosafety cabinet (Model 425–400; NuAire) and adding 100–165 MBq (~ 19 μL) of ^111^In[In]Cl_3_ (BWXT Medical Ltd.) mixed with 38 μL of 0.05 M NH_4_CO_2_CH_3_ buffer, pH 5.5 using a digital pipette and sterile tip. The vial was incubated at RT for 1–2 h, then diluted to a final volume of 2.0 mL by addition of Sodium Chloride Injection, USP. The radiopharmaceutical solution was then drawn up in a 3 mL syringe with an 18G x 1½" needle and the solution sterilized by passing through a 0.22 μm Millex GV filter into a 5 mL sterile glass vial with grey butyl rubber septum and aluminum seal. The integrity of the sterilizing filter was checked by the bubble test. ^111^In[In]-BnDTPA-trastuzumab-NLS injection was assayed in a radioisotope dose calibrator (Capintec Model CRC25R, Ramsey, NJ, USA) and the specific activity calculated by dividing the radioactivity (MBq) by the mass of BnDTPA-trastuzumab-NLS in the kit (5 mg). The specification was 20–33 MBq/mg. The radiopharmaceutical was tested against specifications for pH (5.5–6.5), radiochemical purity (RCP ≥ 90%), radionuclide purity (≥ 99%), appearance (clear, colourless, particle-free) and sterility (retrospective USP Sterility Test). The pH was measured using pH 4.5–7.5 range pH paper (pHydrion^®^). The RCP was measured by ITLC-SG developed in 0.1 M sodium citrate, pH 5.0. Radionuclide purity was confirmed by the COA for the actual lot of ^111^In[In]Cl_3_ (BWXT Medical Ltd.; ≥ 99%; < 0.1% ^114m^In or ^65^Zn). The appearance was inspected by examining the radiopharmaceutical vial against a light or dark background. Lots of ^111^In-BnDTPA-trastuzumab-NLS injection were tested retrospectively for sterility by the USP Sterility Test after decay for at least 60 d. The stability of ^111^In[In]-BnDTPA-trastuzumab-NLS injection stored at 2–8 °C was assessed over 24 h by re-testing against specifications for clarity and RCP (≥ 90%) to establish an expiry.

### Statistical analysis

Statistical significance was determined using an unpaired Student's t test (*P* < 0.05) using GraphPad software Ver. 9.0 for Mac.

## Results

### Raw materials and pharmaceutical buffers

**Fig. 1:**
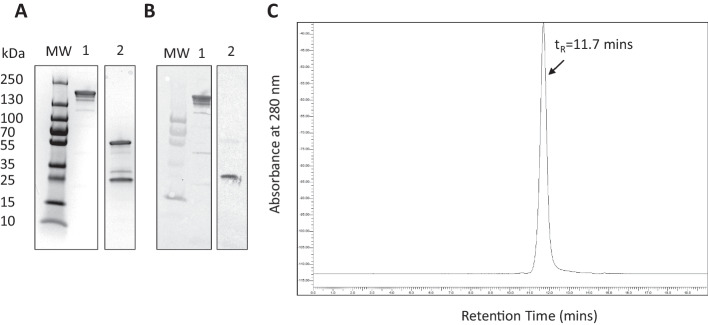
**A** SDS-PAGE analysis of trastuzumab under non-reducing conditions (lane 1) or reducing conditions (DTT, lane 2) on a 4–20% Tris HCl gradient mini-gel. MW: broad range molecular weight standards. The gel was stained with Coomassie R-250 Brilliant Blue. **B** Corresponding Western blot under non-reducing (lane 1) or reducing (lane 2) conditions showing immunopositivity with goat anti-human Fab specific IgG-horseradish peroxidase (HRP) immunoconjugates. Bands were detected by reaction with diamidobenzidine/0.03% H_2_O_2_. **C** SE-HPLC analysis of trastuzumab on a BioSep SEC-s4000 column (Phenomenex, Torrance, CA, USA) eluted with 100 mM NaH_2_PO_4_ buffer, pH 7.0 at a flow rate of 0.8 mL/min with UV detection at 280 nm

Trastuzumab migrated as a single major band on SDS-PAGE under non-reducing conditions corresponding to a protein with the expected MW = 170 kDa and two bands under reducing conditions corresponding to proteins with MW = 50 kDa and 25 kDa, representing the heavy and light chains of trastuzumab (Fig. 1A). These bands were immunopositive on Western blot when probed with goat anti-human Fab-specific horseradish peroxidase (HRP) immunoconjugates (Fig. 1B). SE-HPLC analysis of trastuzumab showed one major peak with retention time (t_R_ = 11.7 min) accounting for > 98% of all peak areas (Fig. 1C). Proton NMR analysis of p-SCN-BnDTPA agreed with the reported ^1^H spectrum (Brechbiel et al. 1986). The COA from the supplier (Macrocyclics) confirmed 94.1% purity. Amino acid composition analysis confirmed the identity of NLS peptides and the COA from the supplier (Bio Basic, Inc.) showed 98.6% purity and a mass spectrum consistent with the expected MW = 1,419 Da. Three lots of 0.1 M NaHCO_3_ buffer, pH 8.2, six lots of 0.1 M Na_2_HPO_4_ buffer, pH 7.3 and three lots of 0.05 M NH_4_CO_2_CH_3_ buffer, pH 5.5 were manufactured. All buffers met specifications for appearance (clear, colourless and particle-free), concentration (expected ± 10%) and pH (expected ± 0.1 units) and passed the USP Sterility Test.

### Kit formulation and quality testing

**Fig. 2:**
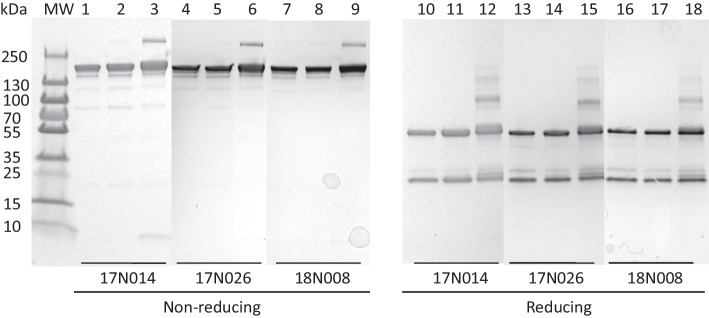
**A** SDS-PAGE analysis under non-reducing conditions of trastuzumab (lanes 1,4,7), BnDTPA-trastuzumab (lanes 2,5,8) and BnDTPA-trastuzumab-NLS in kit lots 17N014, 17N026 and 18N008 (lanes 3, 6, 9). **B.** SDS-PAGE analysis under reducing conditions (DTT) of trastuzumab (lanes 10,13,16), BnDTPA-trastuzumab (lanes 11,14,15) and BnDTPA-trastuzumab-NLS in kit lots 17N014, 17N026 and 18N008 (lanes 12, 15,18). The gel was stained with Coomassie R-250 Brilliant Blue. MW: broad range molecular weight standards

**Fig. 3:**
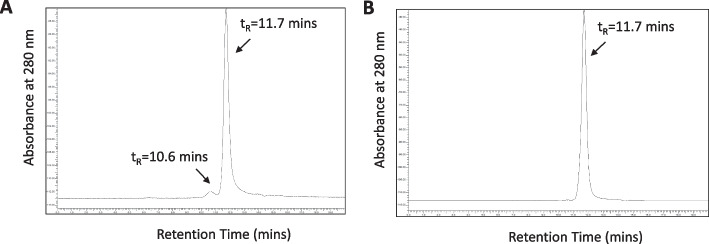
Representative SE-HPLC chromatograms for **A** BnDTPA-trastuzumab-NLS and **B** trastuzumab on a BioSep SEC-s4000 column (Phenomenex, Torrance, CA, USA) eluted with 100 mM NaH_2_PO_4_ buffer, pH 7.0 at a flow rate of 0.8 mL/min with UV detection at 280 nm. Trastuzumab and BnDTPA-trastuzumab-NLS exhibited one major peak with retention time (t_R_ = 11.7 min) but BnDTPA-trastuzumab-NLS exhibited a second peak at t_R_ = 10.6 min. This small second peak may be due to cross-linking of BnDTPA-trastuzumab after reaction with Sulfo-SMCCrequired to introduce maleimide groups for conjugation to the thiol on cysteine in NLS peptides

**Fig. 4:**
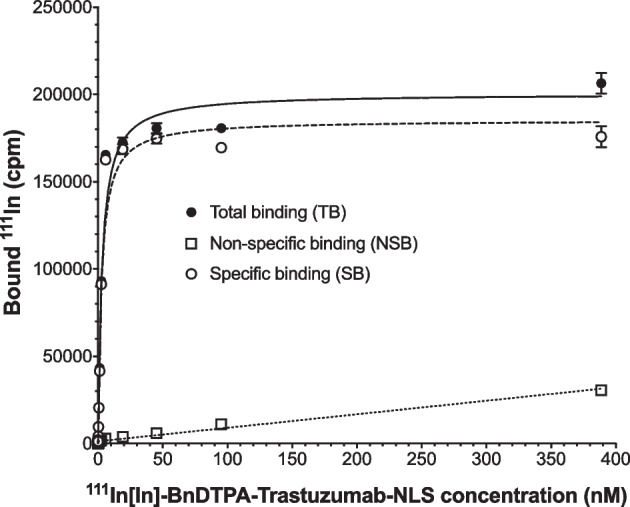
Representative direct (saturation) receptor-binding curve for the binding of ^111^In[In]-BnDTPA-trastuzumab-NLS prepared from kit Lot 18N008 to HER2-positive SK-BR-3 human breast cancer (BC) cells. Shown are the total binding (TB) and non-specific binding (NSB) in the presence of an excess (15.2 μmoles/L) of trastuzumab. The specific binding (SB) was calculated by subtracting NSB from TB. In this representative HER2 binding assay, the K_a_ was 3.8 ± 0.6 × 10^8^ L/mole and the B_max_ was 1.0 ± 0.03 × 10^6^ sites per cell

Three sequential lots of kits (8–9 vials/lot) were manufactured (Lots 17N014, 17N026, 18N008). All lots met specifications (Table 1). Protein concentration ranged from 5.3–5.4 mg/mL. The volume was 1.0 mL and the pH was 6.0 for all lots. BnDTPA substitution ranged from 3.8–5.1 BnDTPA/trastuzumab. SDS-PAGE analysis under non-reducing conditions (Fig. 2A) showed a single major band for trastuzumab (MW = 170 kDa, lanes 1/4/7) and BnDTPA-trastuzumab (MW = 170 kDa, lanes 2,5,8). Analysis of 3 kit lots of BnDTPA-trastuzumab-NLS (lanes 3/6/9) under non-reducing conditions showed a slight upward shift of the major protein band compared to BnDTPA-trastuzumab indicating NLS peptide conjugation. Based on the band shift, we estimated the number of NLS peptides conjugated to BnDTPA-trastuzumab for kit lots 17N014, 17N026 and 18N008 were 3, 2 and 3, respectively (mean ± SD = 2.7 ± 0.4). There was a faint higher MW protein band for BnDTPA-trastuzumab-NLS (MW > 250 kDa) but not for BnDTPA-trastuzumab or trastuzumab, which may represent cross-linked immunoconjugates caused by reaction of BnDTPA-trastuzumab with Sulfo-SMCC to introduce maleimide groups required for subsequent conjugation to NLS peptides. This band accounted for < 8% of all protein bands by gel densitometry. Under reducing conditions, trastuzumab (lanes 10/13/16) and BnDTPA-trastuzumab (lanes 11/14/17) migrated as two major bands (MW = 50 kDa and 25 kDa) corresponding to the heavy and light immunoglobulin chains while the bands for BnDTPA-trastuzumab-NLS (lanes 12/15/18) migrated at slightly higher MW of 55 and 28 kDa, respectively. There was a faint higher MW band (MW = 110 kDa) for BnDTPA-trastuzumab-NLS but not for trastuzumab or BnDTPA-trastuzumab, which may represent reduced cross-linked immunoconjugates following Sulfo-SMCC conjugation. SE-HPLC analysis of a representative lot (17N014) of kits (Fig. 3A) showed one major peak (> 90%) for BnDTPA-trastuzumab-NLS with a t_R_ = 11.7 min and a small secondary peak at t_R_ = 10.6 min (< 10%). This small peak may represent cross-linked immunoconjugates due to reaction with Sulfo-SMCC. Trastuzumab eluted as a single peak with t_R_ = 11.7 min (Fig. 3B). The labeling efficiency of the kits with ^111^In was consistently very high: 99.0%, 98.4% and 98.6% for kit lots 17N014, 17N026 and 18N008, respectively, and met specifications for labeling efficiency (≥ 90%; Table 1). ^111^In[In]-BnDTPA-trastuzumab-NLS injection prepared from the kits exhibited preserved high affinity binding to HER2-positive SK-BR-3 cells (Table 1). The K_a_ and B_max_ values for lots 17N014, 17N026, 18N008 were 6.2 ± 0.9 × 10^8^ L/mol and 0.9 ± 0.03 × 10^6^ sites/cell, 4.6 ± 0.7 × 10^8^ L/mol and 0.9 ± 0.2 × 10^6^ sites/cell and 5.6 ± 1.0 × 10^8^ L/mol and 0.9 ± 0.05 × 10^6^ sites/cell, respectively. A representative radioligand binding curve for ^111^In[In]-BnDTPA-trastuzumab-NLS injection prepared from kit lot 18N008 is shown in Fig. 4. All lots of kits passed the USP Sterility Test and USP Bacterial Endotoxins Test.

**Table 1:** Quality testing results for three sequential lots of kits for the preparation of ^111^In[In]-BnDTPA-trastuzumab-NLS injection

Parameter	Specification	17N014	17N026	18N008
Protein concentration	4.5–5.5 mg/mL	5.3	5.4	5.4
Volume	0.95–1.05 mL	1.0	1.0	1.0
pH	5.5–6.0	6.0	6.0	6.0
Appearance	Clear, pale yellow, particle-free	Passed	Passed	Passed
BnDTPA substitution	2.0–7.0 BnDTPA/trastuzuumab	3.9	3.8	5.1
NLS peptide substitution (SDS-PAGE)	Upward shift in major band for BnDTPA-trastuzumab-NLS compared to BnDTPA-trastuzumab	Passed	Passed	Passed
Purity and homogeneity (SDS-PAGE)	Non-reducing conditions: 1 major band (170 kDa) Reducing conditions: 2 major bands (50 kDa and 25 kDa)	Passed	Passed	Passed
Purity and homogeneity (SE-HPLC)	1 major peak at t_R_ = 11.7 ± 0.2 min	Passed	Passed	Passed
^111^In Labeling efficiency (ITLC)	≥ 90%	99.0	98.4	98.6
HER2 binding (SK-BR-3 cells)	K_a_ = 1.0–8.0 × 10^8^ L/mole; B_max_ = 0.5–2.0 × 10^6^ sites/cell	6.2 ± 0.9 x 10^8^ L/mole; 0.9 ± 0.03 x 10^6^ sites/cell	4.6 ± 0.7 x 10^8^ L/mole; 0.9 ± 0.2 x 10^6^ sites/cell	5.6 ± 1.0 x 10^8^ L/mole; 0.9 ± 0.05 x 10^6^ sites/cell
Sterility	USP Sterility Test	Passed	Passed	Passed
Endotoxins	USP Bacterial Endotoxins Test	Passed	Passed	Passed

### Stability testing of kits and expiry

**Fig. 5:**
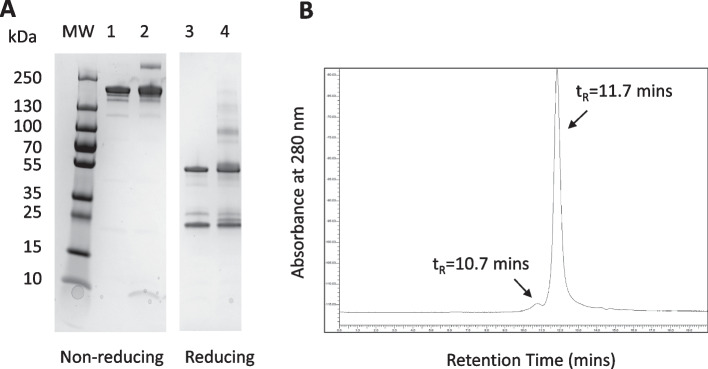
A SDS-PAGE analysis on a 4–20% Tris HCl gradient mini-gel of trastuzumab or BnDTPA-trastuzumab-NLS from kit lot 17N014 after storage at 2–8 °C for 661 d, under non-reducing conditions (lanes 1 and 2, respectively) or reducing (DTT) conditions (lanes 3 and 4 respectively). MW: broad range molecular weight standards. The gel was stained with Coomassie R-250 Brilliant Blue. **B** SE-HPLC analysis of BnDTPA-trastuzumab-NLS from kit lot 17N014 after storage at 2–8 °C for 661 d on a BioSep SEC-s4000 column (Phenomenex, Torrance, CA, USA) eluted with 100 mM NaH_2_PO_4_ buffer, pH 7.0 at a flow rate of 0.8 mL/min with UV detection at 280 nm. BnDTPA-trastuzumab-NLS exhibited one major peak with retention time (t_R_ = 11.7 min) and a second peak at t_R_ = 10.7 min

The stability of kits stored at 2–8 °C was assessed up to 304 d for lot 18N008, 589 d for lot 17N026 and 661 d for lot 17N014 by re-testing against key quality specifications except sterility and endotoxins (Table 2). All lots met specifications for pH, appearance and purity and homogeneity by SDS-PAGE and SE-HPLC (representative lot 17N014 shown in Fig. 5). Protein concentration was slightly higher (5.6–5.9 mg/mL) than specification (4.5–5.5 mg/mL) at extended storage times (304–661 d). However, protein concentration was within specification for kit lot 17N014 up to 127 d (5.4 mg/mL) and kit lot 17N026 up to 139 d (5.5 mg/mL) and only slightly higher than specification for kit lot 18N008 at 129 d (5.6 mg/mL). The labeling efficiency with ^111^In remained high: 97.4%, 97.6% and 98.1% for kit lots 18N008, 17N026 and 17N014, respectively, when stored up to 661 d, 589 d and 304 d, respectively (Table 2). ^111^In[In]-BnDTPA-trastuzumab-NLS Injection prepared from the kits exhibited preserved high affinity specific binding to HER2-positive SK-BR-3 human BC cells (mean ± SD: K_a_ = 3.6 ± 0.4 × 10^8^ L/mol; B_max_ = 1.1 ± 0.1 × 10^6^ sites/cell). Based on these testing results, and to limit the protein concentration exceeding specification, an expiry of 180 d was assigned to the kits stored at 2–8 °C.

**Table 2:** Stability testing results for three sequential lots of kits for the preparation of ^111^In[In]-BnDTPA-trastuzumab-NLS injection

Parameter	Specification	17N014				17N026				18N008			
		9 d ^a^	93 d	127 d	661 d	21 d	97 d	139 d	589 d	16 d	94 d	129 d	304 d
Protein concentration	4.5–5.5 mg/mL	5.3	n.d	5.4	5.6 ^b^	5.4	n.d	5.5	5.6 ^b^	5.4	n.d	5.6 ^b^	5.9 ^b^
pH	5.5–6.0	6.0	6.0	6.0	6.0	6.0	6.0	6.0	6.0	6.0	6.0	6.0	6.0
Appearance	Clear pale yellow, particle-free	Passed	Passed	Passed	Passed	Passed	Passed	Passed	Passed	Passed	Passed	Passed	Passed
Purity and homogeneity	SDS-PAGE: 1 major band (170 kDa) under non-reducing conditions; 2 major bands (50 kDa and 25 kDa) under reducing conditions SE-HPLC: 1 major peak at t_R_ = 11.7 ± 0.2 min	Passed	n.d. ^c^	Passed	Passed	Passed	n.d	Passed	Passed	Passed	n.d	Passed	Passed
^111^In Labeling efficiency	ITLC: ≥ 90%	99.0	98.5	98.3	97.4	98.4	98.5	98.8	97.6	98.6	98.8	98.9	98.1
HER2 binding properties	SK-BR-3 cells: K_a_ = 1.0–8.0 × 10^8^ L/mole; B_max_ = 0.5–2.0 × 10^8^ binding sites/cell	Passed	n.d	Passed	Passed	Passed	n.d	Passed	Passed	Passed	n.d	Passed	Passed

^a^ Storage period at 2–8 °C prior to re-testing against quality specifications

^b^ Slightly outside the specification (4.5–5.5 mg/mL)

^c^ n.d.: Not determined

### ^111^In[In]-BnDTPA-trastuzumab-NLS injection

Fifteen sequential lots of ^111^In[In]-BnDTPA-trastuzumab-NLS injection were prepared (Table 3). All lots passed specifications for specific activity, pH, appearance, radiochemical purity (RCP) and sterility (tested retrospectively). Re-testing after storage at 2–8 °C showed that there was no significant difference between the mean ± SD RCP immediately after preparation (98.4 ± 0.01%) and at 24 h in storage (98.0 ± 0.6%). All lots of ^111^In[In]-BnDTPA-trastuzumab injection remained clear, pale yellow and were particle-free. An expiry of 8 h was assigned to ^111^In[In]-BnDTPA-trastuzumab-NLS injection stored at 2–8 °C, since the product was intended to be administered to a patient within this time period.

**Table 3:** Quality testing results for 15 sequential lots of ^111^In[In]-BnDTPA-trastuzumab-NLS injection

Parameter	Specification	Mean ± SD (*n* = 15)
Specific activity	20–33 MBq/mg	24.0 ± 1.2 MBq/mg
pH	5.5–6.0	6.0 ± 0.0
Appearance	Clear, pale yellow, particle-free	Passed
Radiochemical purity	≥ 90%	98.4 ± 0.01%
Sterility (retrospective)	USP Sterility Test	Passed

## Discussion

We report here the formulation of a kit under GMP conditions for preparing ^111^In[In]-BnDTPA-trastuzumab-NLS injection in pharmaceutical quality suitable for human studies. ^111^In[In]-BnDTPA-trastuzumab-NLS injection prepared from the kit was recently safely administered to 4 patients with HER2-positive BC to trace the uptake of trastuzumab into brain metastases enhanced by MRIg-FUS (Meng et al. 2021). Our report is the first to describe a kit for preparing ^111^In[In]-BnDTPA-trastuzumab-NLS injection under GMP conditions and to our knowledge, is the first to report formulation of a radiopharmaceutical modified with NLS peptides for human administration. To formulate the kit, trastuzumab was first reacted with p-SCN-BnDTPA to introduce BnDTPA chelators to complex ^111^In. There were 3.9, 3.8 and 5.1 BnDTPA/trastuzumab molecule for kit lots 17N014, 17N026 and 18N008, respectively (Table 1). BnDTPA-trastuzumab was then reacted with Sulfo-SMCC to introduce maleimide groups for reaction with the thiol on cysteine on 13-mer peptides [CGYG*PKKKRKV*GG] that harbour the NLS of SV-40 large T-antigen (italics) (Costantini et al. 2008b). NLS conjugation was confirmed by an upward shift in the band for BnDTPA-trastuzumab-NLS compared to BnDTPA-trastuzumab on SDS-PAGE under non-reducing conditions (Fig. 2). Based on the band shift, we estimated that there were 3, 2 and 3 NLS peptides per trastuzumab molecule for kit lots 17N014, 17N026 and 18N008, respectively. This level of NLS peptides is within the range that we previously reported for ^111^In[In]-DTPA-trastuzumab-NLS (2.5–8.4 NLS peptides per trastuzumab molecule) (Costantini et al. 2007). We noted a small percentage (< 8%) of higher MW (> 250 kDa) protein on SDS-PAGE analysis of BnDTPA-trastuzumab-NLS under non-reducing conditions, that was not present in BnDTPA-trastuzumab or trastuzumab. Similarly, a small percentage (< 10%) of a higher MW protein was detected by SE-HPLC analysis of BnDTPA-trastuzumab-NLS (Fig. 3A; t_R_ = 10.6 min) but not for trastuzumab (Fig. 3B). We believe that this represents cross-linked IgG due to reaction of BnDTPA-trastuzumab with Sulfo-SMCC. Sulfo-SMCC incorporates a sulfosuccinimidyl ester that reacts with primary amines and a maleimide functional group that reacts with thiols. Our intent was to react amine groups on BnDTPA-trastuzumab with Sulfo-SMCC and use the introduced maleimide functionality to conjugate BnDTPA-trastuzumab to the NLS peptides by reaction with the thiol on the cysteine in the peptides. However, recombinant monoclonal antibodies may contain a small number of free thiols that could react with the maleimide functional groups and this may be responsible for the small amount of cross-linking of BnDTPA-trastuzumab molecules observed (Metcalfe 2022).

^111^In[In]-BnDTPA-trastuzumab-NLS injection prepared from the kits exhibited high affinity and specific binding to HER2 on SK-BR-3 human BC cells in a direct (saturation) binding assay (K_a_ = 4.6–6.2 × 10^8^ L/mole; B_max_ = 0.9 × 10^6^ binding sites/cell; Table 1 and Fig. 4). The HER2 binding affinity was similar to that previously reported for ^111^In[In]-DTPA-trastuzumab-NLS, measured in a competition receptor-binding assay using SK-BR-3 cells (K_a_ = 1.7–3.2 × 10^8^ L/mole) (Costantini et al. 2007). We previously reported that ^111^In[In]-BnDTPA-trastuzumab exhibited a K_a_ = 3.2 × 10^8^ L/mole (K_d_ = 3.1 × 10^–9^ mol/L) for binding to HER2 on SK-BR-3 cells and B_max_ = 1 × 10^6^ binding sites/cell. (Chan et al. 2020). The product monograph for trastuzumab (Herceptin, Roche) states that the K_d_ for binding to HER2 = 5 × 10^–9^ mol/L (K_a_ = 2 × 10^8^ L/mole) in a cell-based assay (Roche 2021). Thus, the HER2-binding properties of ^111^In[In]-BnDTPA-trastuzumab-NLS were similar to those of ^111^In[In]-BnDTPA-trastuzumab or unmodified trastuzumab.

The kits achieved a LE = 98.4–99.0% after incubation with ^111^In[In]Cl_3_ at RT for 1–2 h, which met specifications (≥ 90%; Table 1). This very high LE was achieved due to extensive purification during kit formulation by ultrafiltration on an Ultra-15 centrifugal filter device (MWCO = 30 kDa) to remove unreacted p-SCN-BnDTPA from BnDTPA-trastuzumab (ultrafiltration 16 times) and subsequently to purify BnDTPA-trastuzumab-NLS from unconjugated NLS peptides (ultrafiltration 15 times). We have previously used ultrafiltration to remove unconjugated BnDTPA from BnDTPA-pertuzumab to formulate a kit for labeling pertuzumab (Perjeta, Roche) with ^111^In that similarly achieved a very high LE = 98% when incubated with ^111^In[In]Cl_3_ for 30 min at RT (Lam et al. 2015). The very high LE (> 98%) for labeling the kits for ^111^In[In]-BnDTPA-trastuzumab-NLS injection with 111–165 MBq of ^111^InCl_3_ suggests that higher amounts of ^111^In[In]Cl_3_ could be added to the kit, while still meeting the LE specification (≥ 90%). Alternatively, the kit formulation could be scaled up for future therapeutic application, for which higher amounts of activity would be required than for SPECT imaging. There have been very few studies of MAE-emitting radiopharmaceuticals for cancer treatment in patients. In one clinical trial, the single amount of ^111^In[In]-DTPA-pentetreotide radiopeptides administered for MAE radiotherapy of somatostatin receptor-positive neuroendocrine tumours in humans ranged from 2,000 to 11,000 MBq (Valkema et al. 2002). ^111^In[In]-DTPA-EGF was administered to patients with EGFR-positive BC in amounts up to 2,290 MBq in a Phase 1 clinical trial of MAE peptide radiotherapy (Vallis et al. 2014). The amount of ^111^In[In]-BnDTPA-trastuzumab-NLS required for RIT of HER2-positive BC in humans may be much lower than ^111^In[In]-DTPA-pentetreotide or ^111^In[In]-DTPA-EGF due to the slower elimination and generally higher tumour uptake of radiolabeled monoclonal antibodies than radiopeptides (Reilly et al. 2000). However, assuming comparable amounts of 2,000–11,000 MBq, this would require scaling the kit formulation from 5 to 70 mg of BnDTPA-trastuzumab-NLS per vial to achieve labeling with ^111^In at the same specific activity (up to 33 MBq/mg). This mass dose of ^111^In[In]-BnDTPA-trastuzumab-NLS is equivalent to 1 mg/kg in a 70 kg human and is lower than the therapeutic doses of trastuzumab, which are a loading dose of 4 mg/kg and maintenance dose of 2 mg/kg (Roche 2021), thus should be safe.

The kits were stable stored at 2–8 °C for up to 661 d, continuing to meet specifications for key quality parameters (Table 2 and Fig. 5). Sterility and endotoxins were not re-tested. Protein concentration was slightly higher (5.6–5.9 mg/mL) than the specification (4.5–5.5 mg/mL; Table 2) after storage of the kits for extended periods (304–661 d). However, the protein concentration remained within the specification for kit lot 17N014 up to 127 d (5.4 mg/mL) and for kit lot 17N026 up to 139 d (5.5 mg/mL) and was only slightly higher than specification for kit lot 18N008 at 129 d (5.6 mg/mL). This higher than expected protein concentration may be due to minor evaporation of 0.05 M NH_4_CO_2_CH_3_ buffer, pH 5.5 within the sealed vial in storage. However, since the kits are unit-dose vials, this slight variance from the specified protein concentration would not be clinically important as the final radiopharmaceutical product will still consist of 5 mg of BnDTPA-trastuzumab-NLS labeled with ^111^In, which is then diluted to a final volume of 2.0 mL by addition of Sodium Chloride Injection, USP. The expiry of the kits was set to 6 months (180 d) stored at 2–8 °C to limit the protein concentration exceeding specification. Fifteen sequential lots of ^111^In[In]-BnDTPA-trastuzumab-NLS injection were prepared from the kits and all lots met specifications (Table 3) for specific activity (20–33 MBq/mg), pH (5.5–6.0), appearance (clear, pale yellow and particle-free), RCP (≥ 90%) and sterility (USP Sterility Test tested retrospectively). ^111^In[In]-BnDTPA-trastuzumab-NLS injection continued to meet specifications for RCP, pH and appearance stored at 2–8 °C for 24 h. The expiry was set to 8 h because it was intended to administer the radiopharmaceutical to patients within this time period. We previously similarly found that kits for preparing ^111^In[In]-BnDTPA-pertuzumab injection were stable for 4 months stored at 2–8 °C and the final radiopharmaceutical was stable for 24 h, stored at 2–8 °C (Lam et al. 2015). ^111^In[In]-BnDTPA-trastuzumab-NLS injection may also be stable for short periods at RT since we detected no degradation of trastuzumab stored at RT or at 2–8 °C for up to 12 h (Chan et al. 2020).

## Conclusions

A kit for the preparation of ^111^In[In]-BnDTPA-trastuzumab-NLS injection was designed and manufactured under GMP conditions. The kit met specifications for pharmaceutical quality and was stable for up to 661 d when stored at 2–8 °C, except for protein concentration which was slightly greater than specification at extended storage times but remained within or only slightly higher than expected at storage times up to 139 d. ^111^In[In]-BnDTPA-trastuzumab-NLS injection prepared from the kit met all quality specifications and was stable for at least 24 h when stored at 2–8 °C. ^111^In[In]-DTPA-trastuzumab-NLS injection is a promising theranostic agent with application for SPECT imaging and MAE RIT of HER2-positive BC.

## Data Availability

All data generated or analyzed during this study are included in this published article.

## Abbreviations

^111^InCl_3_: Indium-111-chloride
ACS: American Chemical Society
ADC: Antibody–drug conjugate
BBB: Blood–brain-barrier
BC: Breast cancer
B_max_: Total density (concentration) of receptors (sites/cell)
CE: Conjugation efficiency
COA: Certificate of analysis
d: Day
Da: Dalton
DNA: Deoxyribonucleic acid
DTPA: Diethylenetriaminepentaacetic acid
DTT: Dithiothreitol
EGF: Epidermal growth factor
EGFR: Epidermal growth factor receptor
EP: European pharmacopoeia
GBq: Gigabecquerel
GMP: Good manufacturing practices
h: Hour
HCl: Hydrochloric acid
HER2: Human epidermal growth factor receptor 2
HRP: Horseradish peroxidase
i.p.: Intraperitoneal
i.v.: Intravenous
IgG: Immunoglobulin type G
ITLC-SG: Instant thin layer-silica gel chromatography
K_a_: Association constant
kDa: Kilo-dalton
keV: Kilo electron volt
LE: Labeling efficiency
LET: Linear energy transfer
M: Molar (concentration)
mAb: Monoclonal antibodies
MAE: Meitner-auger electron
MBq: Megabecquerel
μg: Micro-gram
mg: Milli-gram
min: Minutes
μL: Micro-litre
mL: Milli-litre
μm: Micro-metre
mmol: Milli-moles
μmoles: Micro-moles
MRI: Magnetic resonance imaging
MRIg-FUS: MRI guided focused ultrasound
MW: Molecular weight
MWCO: Molecular weight cut-off
Na_2_HPO_4_: Sodium phosphate
NaHCO_3_: Sodium bicarbonate
NaOH: Sodium hydroxide
NH_4_CO_2_CH_3_: Ammonium acetate
NLS: Nuclear translocation sequence
nm: Nano-metre
nmoles: Nano-moles
NMR: Nuclear magnetic resonance
NPC: Nuclear pore complex
NSB: Non-specific binding
PBS: Phosphate-buffered saline
p-SCN-Bn-DTPA: 2-(4-Isothiocyanatobenzyl)-diethylenetriaminepentaacetic acid
PVDF: Polyvinylidene fluoride
RCP: Radiochemical purity
RIT: Radioimmunotherapy
RT: Room temperature
SB: Specific binding
s.c.: Subcutaneous
SD: Standard deviation
SDS-PAGE: Sodium dodecyl sulfate–polyacrylamide gel electrophoresis
SE-HPLC: Size-exclusion high performance liquid chromatography
SPECT: Single photon emission computed tomography
Sulfo-SMCC: Sulfosuccinimidyl 4-(N-maleimidomethyl)cyclohexane-1-carboxylate
TB: Total binding
TKI: Tyrosine kinase inhibitor
t_R_: Retention time
USP: United States Pharmacopeia
UV: Ultra-violet
× g: Gravitational centrifugal force
